# Training Program for the Use of Magnification Loupes in Dentistry

**DOI:** 10.1111/eje.70088

**Published:** 2025-12-31

**Authors:** Júlia Margato Pazos, Jessica Katarine de Abreu Silva, Patricia Petromilli Nordi Sasso Garcia

**Affiliations:** ^1^ Department of Social Dentistry, School of Dentistry São Paulo State University (Unesp) Araraquara São Paulo Brazil; ^2^ Department of Restorative Dentistry, School of Dentistry São Paulo State University (Unesp) Araraquara São Paulo Brazil

**Keywords:** dental students, loupes, magnification, training program

## Abstract

**Introduction:**

Although magnification loupes offer well‐documented advantages, their initial adaptation can be challenging, often deterring widespread adoption. Without sufficient training during this critical period, students may feel inadequately prepared, limiting their ability to benefit from loupes and contributing to reluctance in their use. To address this issue, this study designed and evaluated a training program focused on the use of Galilean loupes, aiming to enhance the ergonomic posture of dental students.

**Methods:**

Thirty‐one second‐year dental students were divided into an experimental group and a control group. Response variables were compliance with ergonomic posture requirements, neck angular deviation, muscle contraction of the back and neck and self‐reported perception of pain/discomfort. The independent variables were training program and timing of evaluation (T0, before the program; T1, 1 week after the program). Data analysis used mixed repeated‐measures ANOVA and nonparametric mixed repeated‐measures ANOVA (*α* = 5%).

**Results:**

For posture compliance, the experimental group showed improved scores at T1 (*p* = 0.044), while the control group showed no change. Angular deviation was smaller at T1. No significant interaction was found for muscle contractions. Pain/discomfort perception significantly decreased over time (*p* = 0.006).

**Discussion:**

The training program significantly improved students' compliance to ergonomic posture requirements, though it had no measurable effect on the other variables assessed. These findings are noteworthy, as they indicate that targeted training in the use of magnification loupes can effectively improve students' compliance to ergonomic posture standards. Notably, changes in neck angular deviation and reported pain or discomfort were associated with the timing of assessment rather than the training itself. This suggests that the use of magnification loupes may contribute to reducing angular deviation in the neck as students gain confidence and become more accustomed to magnification. Overall, the training program demonstrated a clear benefit in supporting ergonomic compliance among dental students.

## 1 Introduction

Several strategies have been proposed to improve the compliance of dental students with ergonomic posture requirements and prevent the development of musculoskeletal disorders [1, 2]. One such strategy is the use of magnification, which offers several benefits, notably improving the working posture and preventing musculoskeletal injuries [3, 4, 5, 6, 7].

Despite the numerous benefits of magnification loupes, the initial adaptation period poses a significant barrier to their widespread adoption [8, 9, 10]. The lack of adequate training during this period can leave students feeling unprepared, hindering their ability to fully benefit from the advantages of magnification loupes, and as a result, fostering resistance to their use [10, 11].

According to Alhazzazi et al. [12] and Braga et al. [3], the benefits of magnification become apparent only after adequate training and skill development. To address the initial adaptation barrier, a training program should be introduced during the preclinical phase [8, 11, 12, 13, 14]. Additionally, the use of magnification loupes in preclinical training can enhance the development of psychomotor skills and boost students' confidence during dental procedures [11]. However, improper use of magnification loupes can lead to optical and postural issues [8].

The development and validation of a training program for the use of magnification loupes among dental students could facilitate their integration into clinical practice, helping to understand the influence of environmental and behavioural factors on their use. To the best of our knowledge, no previous study has evaluated such programs. Therefore, this study aimed to create and assess the impact of a training program focusing on the use of Galilean loupes, specifically targeting the ergonomic posture of dental students during preclinical training.

## 2 Materials and Methods

### 2.1 Study Design

This experimental study was approved by the Research Ethics Committee of UNESP, School of Dentistry, Araraquara, Brazil (CAAE Registry No. 52499921.7.0000.5416). Written informed consent was obtained by an independent researcher from all participants. The response variables were: compliance with ergonomic posture requirements, assessed using the Compliance Assessment of Dental Ergonomic Posture Requirements (CADEP) [15]; angular deviation of the neck, measured with the Software for Postural Assessment version 0.69 (Laboratory for Biomechanics and Motor Control Federal University of ABC (UFABC), São Bernardo do Campo, São Paulo, Brazil. Available in: http://pesquisa.ufabc.edu.br/bmclab/sapo/), muscle contraction in the back and neck, assessed via thermography; self‐reported perception of pain/discomfort in the cervical and upper back region. The independent variables were the application of the preclinical training program for work with magnification at two levels (with training, experimental group; without training, control group) and the time of evaluation of the response variables at two levels (T0, before the application of the training; T1, 1 week after the application of the program). Thirty‐one second‐year undergraduate students from the São Paulo State University (Unesp), School of Dentistry of Araraquara agreed to participate in the study. To assess the response variables, all students first performed class I cavity preparation using a magnification loupe without prior training (T0). They were then randomly assigned to either the control or experimental group. The experimental group received training on the use of magnification loupes, whereas the control group did not. One week after T0, all students performed another class I cavity preparation using a magnification loupe (T1). The control group simulated a typical dental course scenario in which students used magnification loupes without prior training. Additionally, T0 and T1 measurements from the control group provided data on changes in response variables that could be attributed solely to the passage of time or repetition.

### 2.2 Magnification Loupes

**FIGURE 1:**
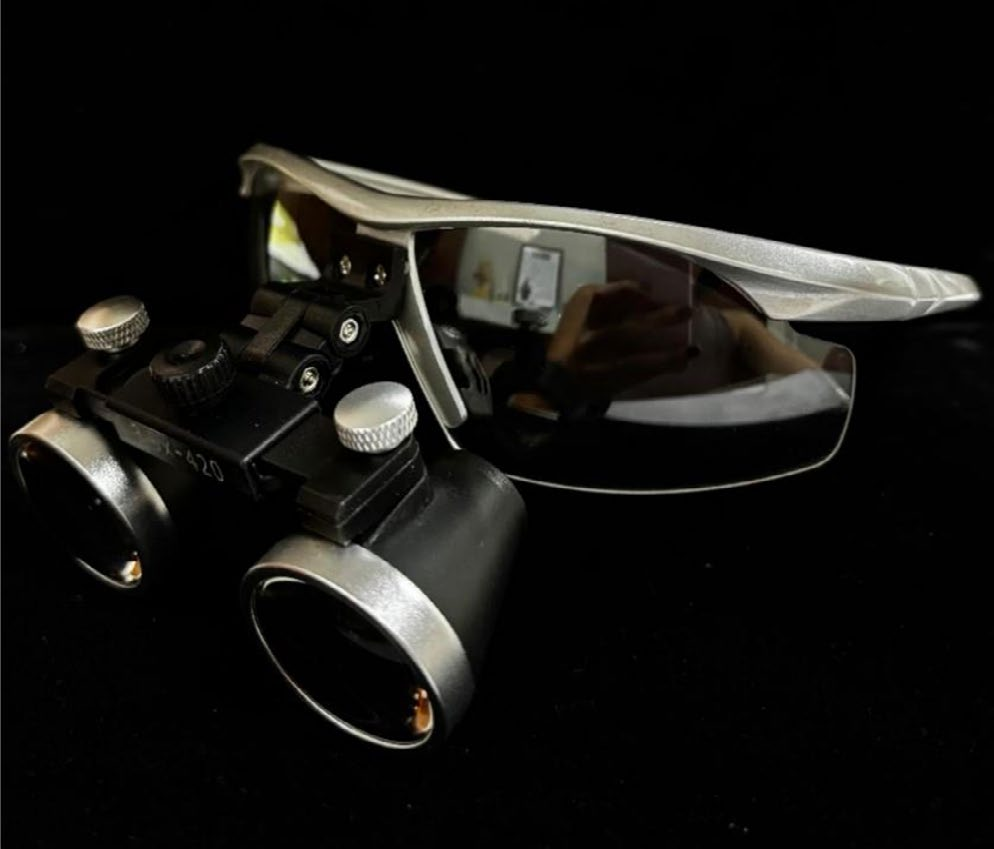
Galilean system binocular loupes with 3.5× magnification.

To perform the procedures proposed in the training, Galilean binocular loupes with 3.5× magnification were used and mounted on frames with a flip‐up system (Nagano, São Paulo, Brazil), allowing adjustment for interpupillary distance and declination angle (Figure 1).

### 2.3 Cell Phone Camera

Each student used their own cell phone camera, which was calibrated for zooming using a tool developed by Rahman and Henderson [16]. This tool includes a printed ‘calibration grid’ and ‘calibration ruler’ to standardise the camera zoom at levels ranging from 1× to 5×.

**FIGURE 2:**
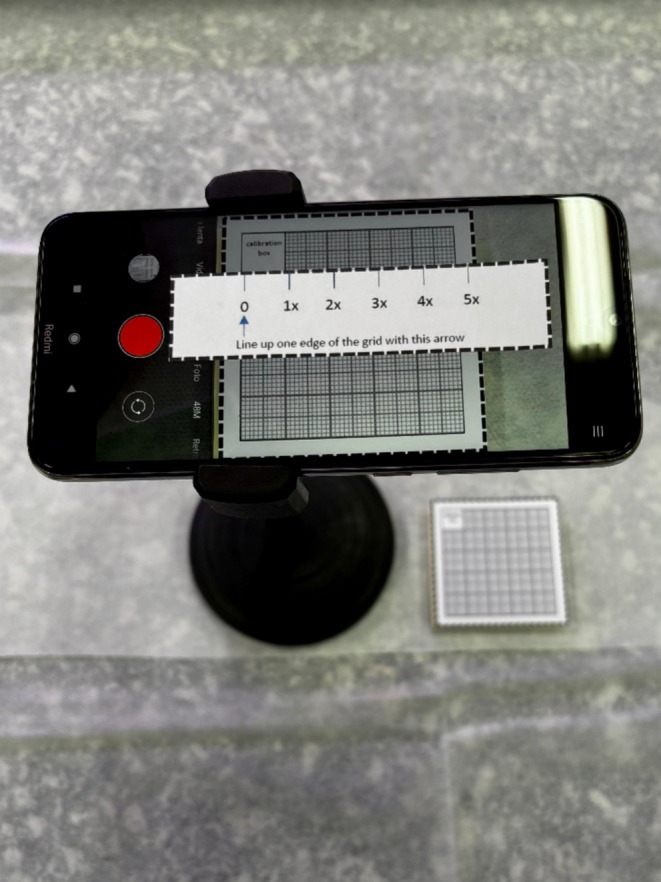
Calibration of the cell phone camera.

The calibration grid features a white box in the upper‐left corner that is visible through the cell phone camera. A calibration ruler placed on the screen was used to align the image of the box to the desired magnification level. The calibration process follows three simple steps: (1) position the cell phone on a stable surface, 7–12 cm from the calibration grid, with the calibration ruler beneath the screen; (2) open the camera in video mode and align the edge of the calibration box image with the ‘0’ point on the ruler; (3) adjust the cell phone's zoom until the width of the image matches the desired magnification level indicated on the ruler (Figure 2).

### 2.4 Program for the Use of Magnification Loupes

The proposed program was designed based on the recommendations of Garcia et al. [17] and implemented in a staggered format, divided into two parts: the first focused on extraoral activities, and the second focused on intraoral activities. The program was structured into five phases, each conducted on a separate day and lasting 1–3 h, totalling 13 h of training (Table 1). Each phase had distinct objectives and progressively increased difficulty.

**TABLE 1:** Phases of the training program.

Phase	Exercise	Region	Magnification	Description	Duration
1	Theoretical	—	—	Theoretical class ‘Magnification: principles of use and development of motor skills’	1 h
2	Practical	Extraoral	Cellphone camera	Training: (a) in the ‘Training Notebook’ drawing a continuous line along the path defined by the double lines of geometric figures; (b) on the ‘Training Plate’ with micromotor and contra‐angle, performing the preparations, deepening the bur at the points marked in black	3 h
3	Practical	Extraoral	Magnification loupe	Training: (a) in the ‘Training Notebook’ drawing a continuous line along the path defined by the double lines of geometric figures; (b) on the ‘Training Plate’ with micromotor and contra‐angle, performing the preparations, deepening the bur at the points marked in black	3 h
4	Practical	Intraoral	Cellphone camera and magnification loupe	Training: (a) location of red targets positioned on the lingual surface of the upper posterior teeth; (b) transfer of the targets located in exercise ‘a’ to the lingual surface of the tooth on the opposite side	3 h
5	Practical	Intraoral	Cellphone camera and magnification loupe	Training: (a) performing cavity preparation in the upper first molar on the operator's working side	3 h

**FIGURE 3:**
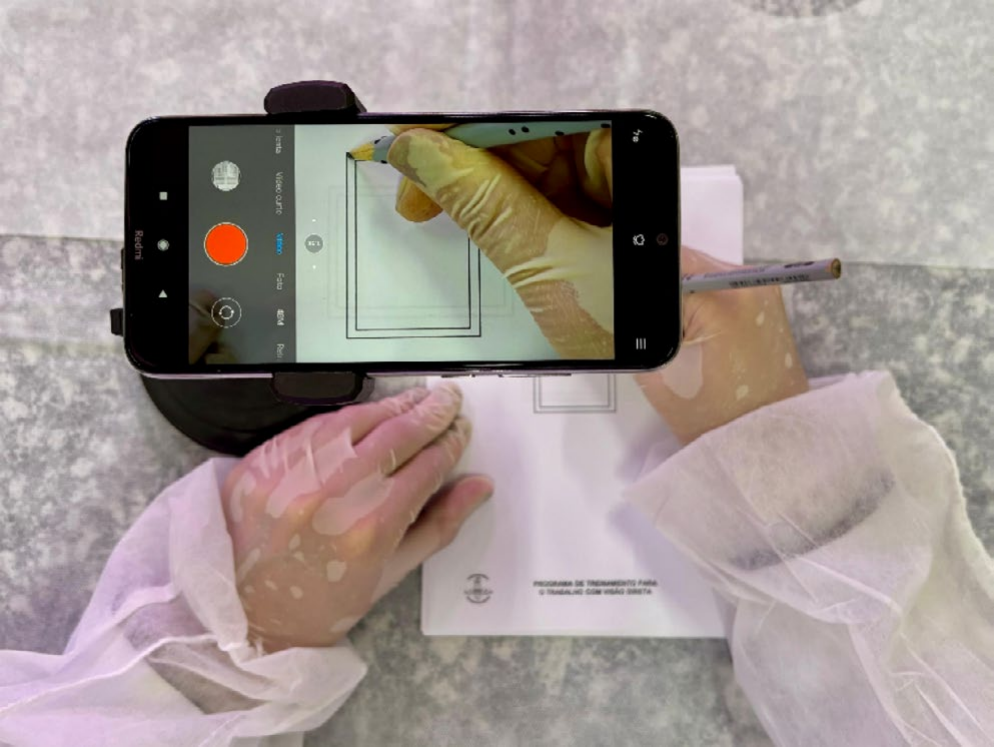
Extraoral exercise of tracing lines in the training notebook.

In phase 1, students attended a 1‐h theoretical class where they learned how to use a magnification loupe and develop manual dexterity with magnification. In phase 2, they began extraoral activities using a cell phone camera positioned on a support with its zoom function previously calibrated [16]. The training started with a notebook containing 10 figures, and the students were required to follow the exact sequence, as presented (Figure 3). The task involved drawing a continuous line with a black pencil along the path defined by the double lines in each figure. Students were required to avoid touching or crossing the lines to ensure that there was uninterrupted tracing.

**FIGURE 4:**
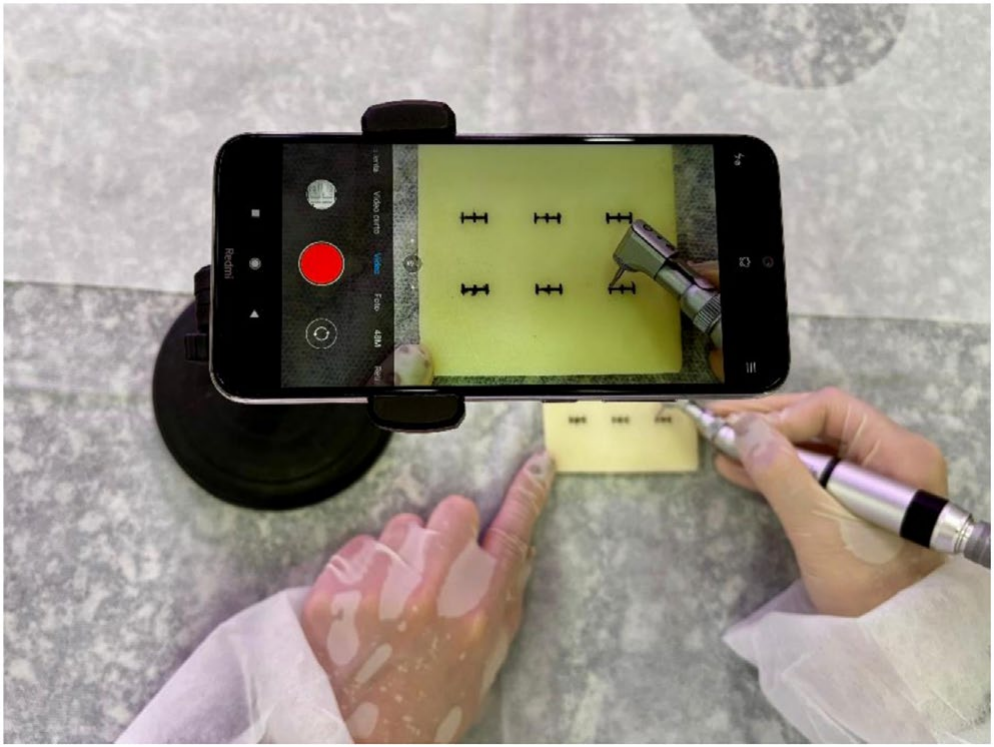
Extraoral exercise simulating class I cavity preparation on a training plate.

After completing the training notebook, students worked on an acrylic plate with six simulated class I cavity preparations on a workbench. Using a low‐speed contra‐angle and a 1014 diamond bur, the participants were instructed to perform the preparations, deepening the bur at the points marked in black on the training plate (Figure 4).

**FIGURE 5:**
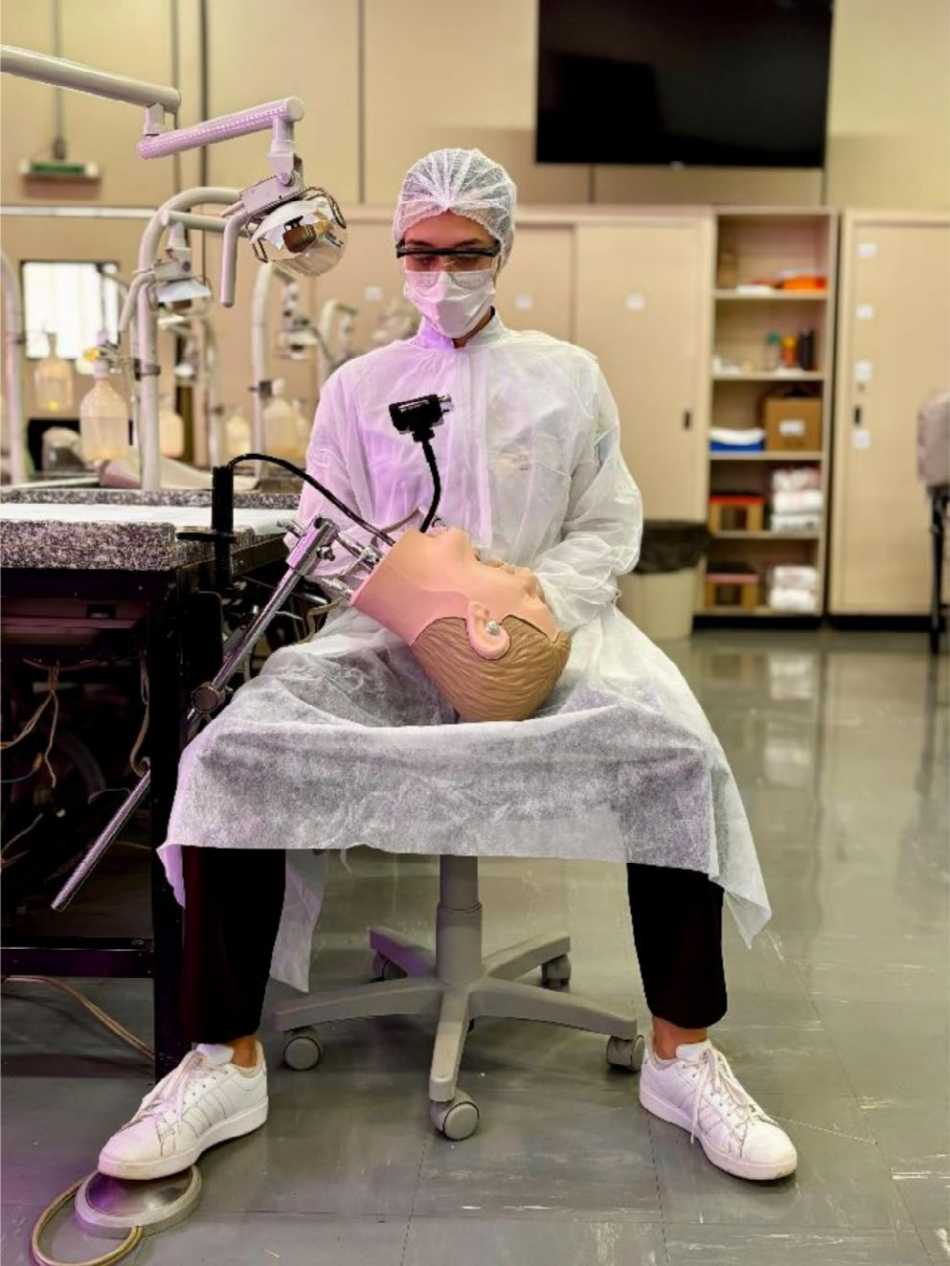
Positioning of the cell phone holder and the student during intraoral exercises using the cell phone camera.

**FIGURE 6:**
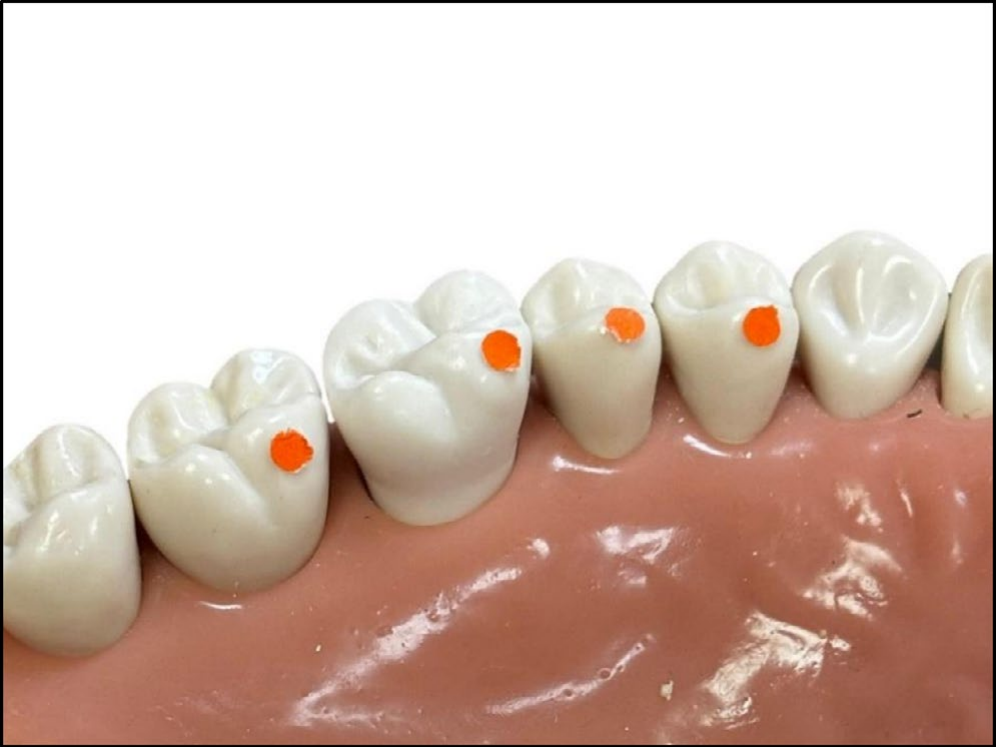
Targets placed on the lingual surface of the upper posterior teeth of a dental mannequin.

In phase 3, the same activities as in phase 2 were performed but using a magnification loupe. In phase 4, intraoral exercise was introduced. The students were instructed to position the cell phone on a support with the camera open and calibrated over the mouth of the dental phantom head, ensuring that they sat comfortably on the stool (Figure 5). Following the methodology adapted from Diesen et al. [18], the students should view the oral cavity exclusively through the cell camera and locate and focus on four circular targets, each 3 mm in diameter, randomly placed on the palatal surface of the upper posterior teeth of a dental mannequin (Figure 6). After locating the targets, the student, using clinical tweezers, transferred each target to the palatal surface of the upper posterior tooth on the opposite side while maintaining focus on the object. This procedure was repeated using a loupe.

**FIGURE 7:**
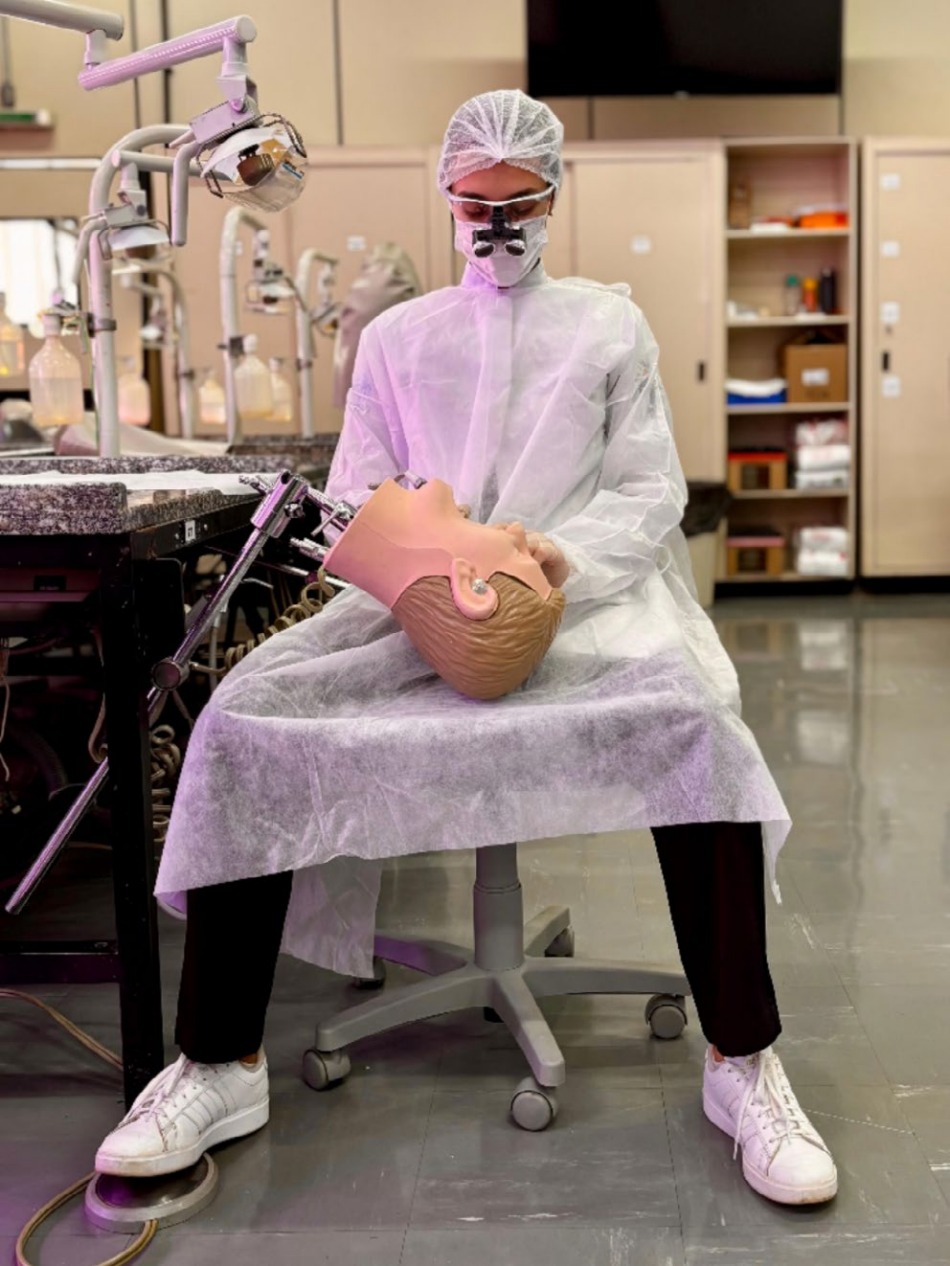
Performing cavity preparation using the magnification loupe.

In phase 5, the students were instructed to position the cell phone on the support, with the camera open and calibrated over the mouth of the patient simulator, and to sit comfortably on the stool. Using a low‐speed contra‐angle with a 1014 spherical bur, the student performed cavity preparation on the upper first molar of the working side, following the tooth's anatomy, and observed only the image on the cell phone. After completing cavity preparation, the prepared tooth was removed and replaced with an intact tooth, allowing the student to perform a new cavity preparation using a loupe (Figure 7).

### 2.5 Evaluation of the Training Program for the Use of Magnification

The effect of the training program on the use of magnification was assessed based on four dependent variables: compliance with ergonomic posture requirements, angular deviation of the neck, muscle activity (measured using thermography) and perception of cervical pain/discomfort. Evaluations were conducted at two time points: T0, when all students performed class I cavity preparation with a magnification loupe without prior training, and T1, after the training program was applied only to the experimental group, when all students (both experimental and control groups) performed class I cavity preparation with a magnification loupe.

For the evaluations, students were instructed to perform a class I cavity preparation for the composite resin using a 1014 diamond bur at low speed. The preparations were performed in the same environment as that of the training program. A dental mannequin (Manequins Odontológicos Marília—Marília, São Paulo, Brazil) with artificial resin teeth designed for cavity preparation at the laboratory level was used. The teeth that had been prepared prior to the training program were removed and replaced with intact resin teeth for post‐training evaluation.

### 2.6 Recording of Working Postures

Working postures were recorded during cavity preparation by filming the entire procedure using digital cameras (GoPro Hero 4—GoPro Inc., San Mateo, CA, USA) mounted on tripods to capture both the lateral and frontal views of the operator. Two filming positions were used to ensure comprehensive visualisation of all body parts to be evaluated. The filming points were determined based on a previous study by Pazos et al. [6].

### 2.7 Compliance With Ergonomic Posture Requirements

Compliance with ergonomic posture requirements was assessed using the modified compliance assessment of dental ergonomic posture requirements (CADEP) proposed by Garcia et al. [15]. The modified CADEP evaluates the following postural aspects: back inclination; back positioning in relation to lumbar support; use of the stool seat; dental chair position; mannequin head position; chair height in relation to the operator's leg beneath the backrest; reflector position; working distance between the mannequin's mouth and the operator's eyes; and the positions of the right and left arms. The assessment focused on the most frequently adopted postures during each procedure and was conducted by a calibrated researcher (ρ = 0.789). Each item of the CADEP was evaluated and categorised as adequate (1 point), partially adequate (0.5 point), or inadequate (0 point). At the end of the evaluation, the scores for all items were summed up, totaling a maximum of 10 points.

### 2.8 Angular Deviation

**FIGURE 8:**
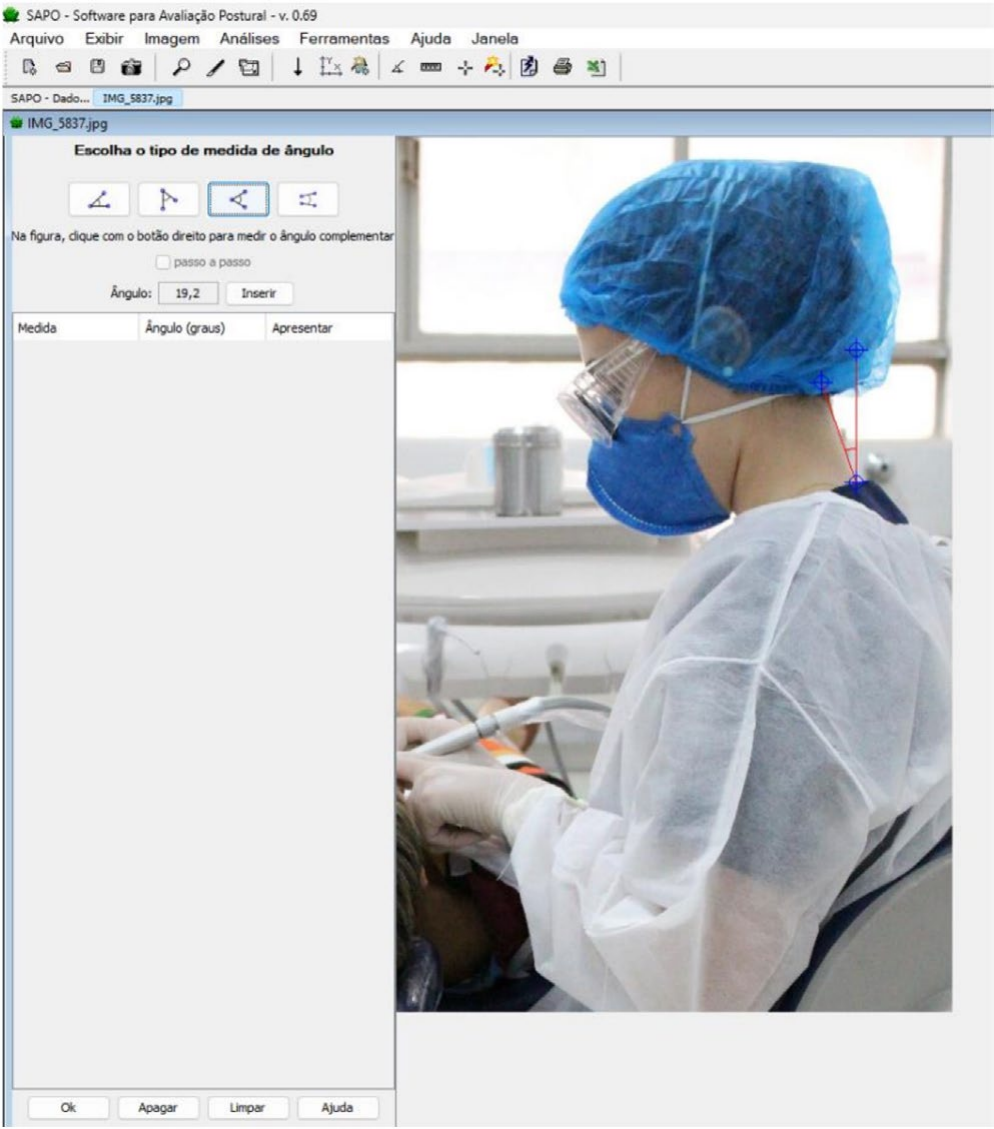
Angular deviation measurement.

The lateral view of the participant's neck during cavity preparation was used to measure angular deviation from the neutral neck position. This measurement was conducted using the Software for Postural Assessment version 0.69. The software first placed a reference point on the C7 vertebra, from which a vertical line was drawn. The second line indicates the inclination of the neck. The software then calculates the angle formed by these three points, providing a numerical value for the angular deviation (Figure 8).

### 2.9 Thermography

Thermal images were acquired using a FLIR C3x infrared camera (FLIR Systems, Wilsonville, OR, USA) with Thermidas version 1.0. The camera features a 320 × 240 element array detector, a thermal sensitivity < 0.10°C and an accuracy of ±2°C at 25°C. It was calibrated by the manufacturer and had a skin emissivity value of 0.98. Thermal images were captured with the participant standing in a room with an ambient temperature ranging from 22°C to 24°C. The camera was positioned 1 m from the participant [19]. Following the guidelines adapted from Pedreira et al. [20], images were obtained at two distinct time points: T0 and T1. At both time points, an image was captured prior to cavity preparation to serve as a control.

### 2.10 Self‐Reported Perception of Pain/Discomfort

Each participant's self‐reported perception of cervical pain/discomfort was assessed using a visual analog scale, which consists of a 10‐cm line with endpoints indicating the minimum and maximum levels of pain.

After each cavity preparation, the participants marked a vertical line on the visual analog scale to indicate the level of cervical pain or discomfort experienced during the procedure. The left endpoint of the scale (point 0) represented no pain or discomfort, whereas the right endpoint (point 10) indicated maximum pain or discomfort. The recorded mark was then assigned a numerical value by measuring the distance from point 0 to the participant's mark using a millimetre ruler.

### 2.11 Statistical Analysis

Descriptive statistical analyses were conducted, followed by verification of the assumptions of normality and sphericity. The effects of the training program (experimental group vs. control group, independent measures) and time (T0 vs. T1, repeated measures) on compliance with ergonomic posture, neck angular deviation, and muscle contraction were assessed using mixed repeated‐measures analysis of variance (ANOVA) with a Bonferroni post‐test. For the perception of pain/discomfort, a nonparametric mixed repeated‐measures ANOVA with a Bonferroni post‐test was used. A significance level of 5% was used for all analyses.

## 3 Results

The mean, standard deviation, and summary of the mixed repeated‐measures ANOVA of compliance with the ergonomic posture requirements of dental students are presented in Table 2.

**TABLE 2:** Mean, standard deviation and summary of the mixed repeated‐measures ANOVA of compliance with ergonomic posture requirements of dental students.

Group	Moment	
	T0	T1
Experimental	7.20 ± 1.29^Aa^	8.97 ± 0.72^Ba^
Control	7.00 ± 1.41^Aa^	7.62 ± 1.37^Ab^

*Note:* Mixed repeated‐measures ANOVA: moment (*F* = 19.404, *p* < 0.001, π = 0.989), group (*F* = 4.782, *p* = 0.037, π = 0.561) and moment × group (*F* = 4.422, *p* = 0.044, π = 0.529). For the Bonferroni post hoc test, the same letters indicate statistical similarity (capital letters, columns; lowercase letters, rows).

For compliance with ergonomic posture requirements, a significant interaction was observed between the factors group and moment (*p* = 0.044), with a higher score at T1 for the experimental group and no significant difference between T0 and T1 for the control group. The experimental group showed greater compliance with ergonomic posture requirements than the control group at T1.

The mean, standard deviation, and summary of the mixed repeated‐measures ANOVA of the angular deviation of the neck in the dental students are shown in Table 3.

**TABLE 3:** Mean, standard deviation and summary of the mixed repeated‐measures ANOVA of the angular deviation of the neck of the dental students.

Group	Moment	
	T0	T1
Experimental	52.97 ± 13.55	40.68 ± 11.88
Control	57.49 ± 15.82	47.92 ± 12.95

*Note:* Mixed repeated‐measures ANOVA: moment (*F* = 76.986, *p* < 0.001, π = 1.000), group (*F* = 1.535, *p* = 0.225, π = 0.224) and moment × group (*F* = 1.196, *p* = 0.283, π = 0.185).

**FIGURE 9:**
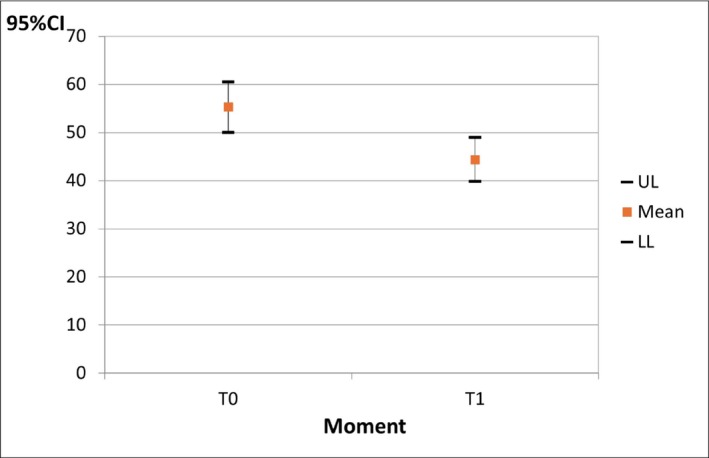
95% CI of the angular deviation of the neck, according to the moment. Mixed repeated‐measures ANOVA: *F* = 76.986, *p* < 0.001, π = 1.000. CI, confidence interval; UL, upper limit; LL, lower limit.

There was no significant interaction for the factors moment and group (*p* = 0.283), only for the factor moment when considered in isolation (*p* < 0.001); for this factor, a 95% confidence interval was constructed (Figure 9).

The angular deviation of the neck was smaller at T1 than at T0 regardless of the application of training.

The mean, standard deviation, and summary of the mixed repeated‐measures ANOVA of contraction of the sternocleidomastoid and ascending trapezius muscles bilaterally are shown in Table 4.

**TABLE 4:** Mean, standard deviation and summary of the mixed repeated‐measures ANOVA of muscle contraction of the sternocleidomastoid and ascending trapezius bilaterally.

Group	Moment	
	T0	T1
Right sternocleidomastoid		
Experimental	0.24 ± 2.54	0.67 ± 2.21
Control	0.31 ± 2.29	1.02 ± 1.73
Left sternocleidomastoid		
Experimental	0.57 ± 2.28	0.43 ± 1.85
Control	0.07 ± 2.47	0.79 ± 1.54
Right trapezius		
Experimental	0.59 ± 3.57	0.35 ± 2.50
Control	0.43 ± 1.55	0.22 ± 1.64
Left trapezius		
Experimental	0.06 ± 2.84	0.17 ± 2.46
Control	0.59 ± 2.11	0.06 ± 1.69

*Note:* Mixed repeated‐measures ANOVA: right sternocleidomastoid: moment (*F* = 2.501, *p* = 0.125, π = 0.334), group (*F* = 0.548, *p* = 0.465, π = 0.111) and moment × group (*F* = 0.036, *p* = 0.851, π = 0.054); left sternocleidomastoid: moment (*F* = 0.864, *p* = 0.360, π = 0.146), group (*F* = 2.564, *p* = 0.120, π = 0.340) and moment × group (*F* = 0.392, *p* = 0.536, π = 0.093); right trapezius: moment (*F* = 0.157, *p* = 0.695, π = 0.067), group (*F* = 0.049, *p* = 0.826, π = 0.055) and moment × group (*F* = 0.001, *p* = 0.977, π = 0.050); left trapezius: moment (*F* = 0.501, *p* = 0.485, π = 0.105), group (*F* = 0.352, *p* = 0.557, π = 0.089) and moment × group (*F* = 0.081, *p* = 0.778, π = 0.059).

No significant interaction was observed between the factors moment and group for any of the muscles evaluated, nor were there significant effects when the factors were considered separately.

**FIGURE 10:**
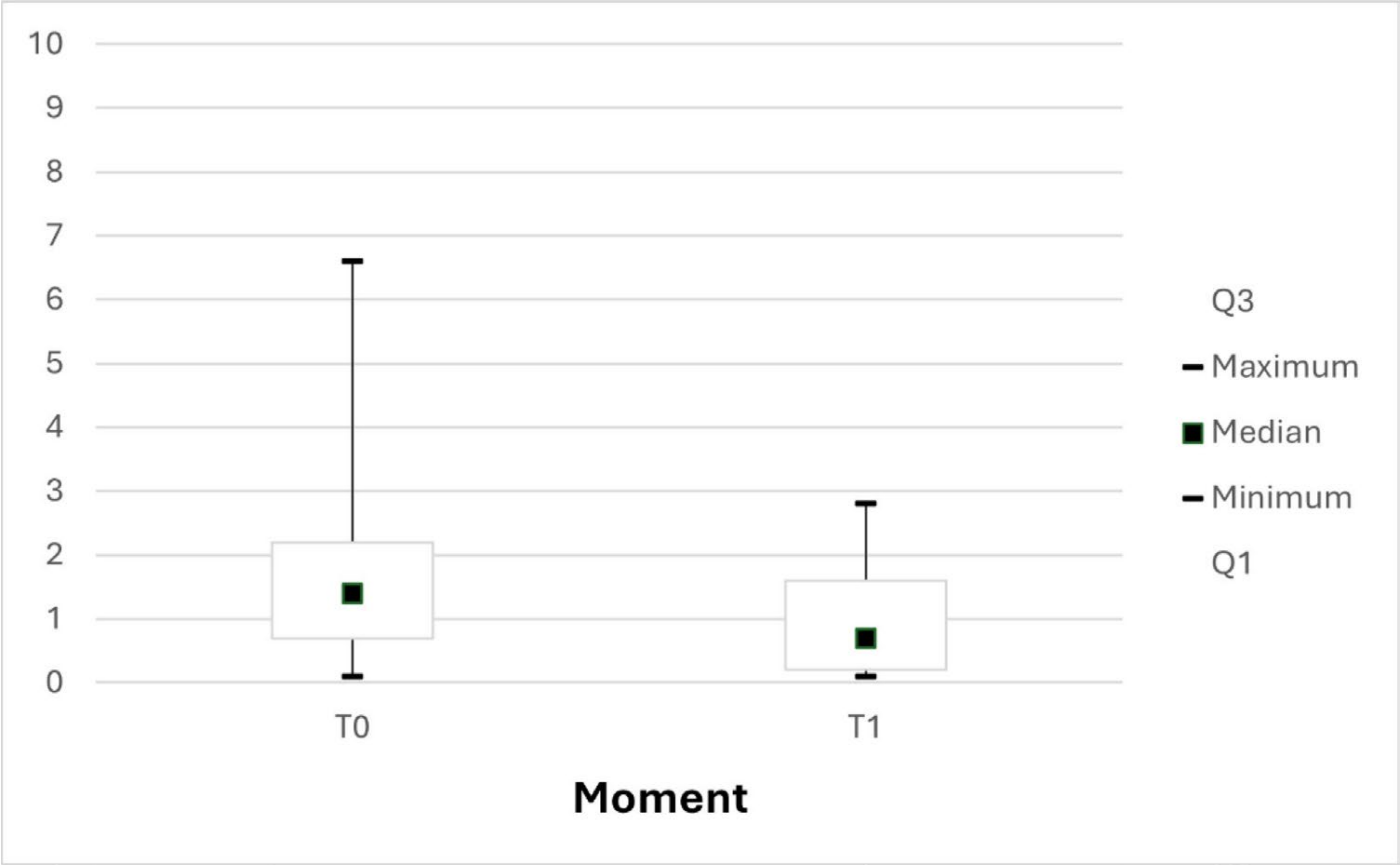
Box‐plot of students' perception of pain/discomfort. Non‐parametric mixed repeated‐measures ANOVA: Moment: *F* = 8.974, *p* = 0.006, *ɳ* ^2^ = 0.236; moment × group: *F* = 0.443, *p* = 0.511, *ɳ* ^2^ = 0.015; group: *F* = 3.751, *p* = 0.063, *ɳ* ^2^ = 0.115.

As the students' perception of pain/discomfort did not meet the assumption of normality, a nonparametric mixed repeated‐measures ANOVA was conducted. A box‐plot of the data, along with a summary of the nonparametric mixed repeated‐measures ANOVA, is presented in Figure 10.

There was no statistical significance in the interaction between the factors moment and group, only for the factor moment when considered in isolation. A lower perception of pain and discomfort was observed in the T1 group.

## 4 Discussion

This study developed and assessed the impact of a training program that focused on using Galilean loupes to improve the ergonomic postures of dental students during preclinical training. The program was implemented using Galilean loupes at 3.5× magnification, based on the premise that training with the highest available magnification would help students transition more easily to lower magnification. Previous studies have suggested that the magnification level did not significantly affect the variables under analysis [5, 21].

The training program was found to have a positive impact on student compliance with ergonomic posture requirements but did not influence the other variables studied. These results are significant, as they demonstrate that training in the use of magnification loupes can enhance students' compliance with ergonomic posture guidelines. This supports the hypothesis that proper training leads to postural benefits when using magnification [3, 12]. In contrast, magnification loupes alone did not lead to postural improvements in students who did not undergo training. This finding contrasts with several studies that reported improved working posture when using loupes compared with working with the naked eye [3, 5, 6, 7, 8, 22].

Another important finding was that angular deviation of the neck and perception of pain/discomfort were influenced solely by the timing of the assessment rather than by the training itself. This suggests that the use of magnification loupes may contribute to reducing angular deviation in the neck as students gain confidence and become more accustomed to magnification [3, 11, 23]. This, in turn, may help alleviate the feelings of pain and discomfort.

Neither training nor assessment time had a significant effect on neck muscle contraction. This could be due to the limited time each student spent performing the cavity preparations, as each student completed only one preparation for each loupe type. It is likely that the prolonged use of magnification loupes during extended work periods has a more substantial impact on muscle activity, potentially reducing contraction and fatigue [2].

At the School of Dentistry of Araraquara—UNESP, students are taught ergonomic posture requirements to help preserve musculoskeletal health throughout their careers [15, 24]. These guidelines emphasise the proper positioning of both the patient and operator, as well as the correct alignment of the operator's head, neck, trunk, arms, forearms, hips, thighs, legs and feet during dental procedures. In this context, the results of the present study were positive. Although the training program did not significantly affect angular deviation, pain/discomfort perception, or muscle contraction in a single region of the body (neck), it did improve students' compliance with ergonomic posture requirements. This compliance reflects a more comprehensive approach to maintaining proper posture throughout the body.

One limitation of this study is that only one procedure was performed at each data collection time point, which limited the insights gained through thermography. Future research could address this by applying training to a larger and more diverse sample, including students from different years of the program. This would enable a more comprehensive assessment of how the variables behave over time and help identify the optimal point for implementing training. Additionally, it would be valuable to consider other variables such as procedure quality, working distance and muscle activity. Despite this limitation, to our knowledge, no other study has developed or evaluated the impact of training that specifically focused on the use of magnification. Therefore, this study fills an important gap in the literature by highlighting its main strengths.

## 5 Conclusion

Training in the use of magnification loupes has a positive effect on compliance with ergonomic posture requirements of students.

## Funding

This work was supported by the São Paulo Research Foundation (FAPESP) under Grants #2021/12031‐4 and #2021/13408‐4; Coordenação de Aperfeiçoamento de Pessoal de Nível Superior – Brazil (CAPES) – Grant 001.

## Conflicts of Interest

The authors declare no conflicts of interest.

## Data Availability

The dataset supporting the findings of this study is publicly available at the following DOI: https://doi.org/10.17632/nb7ctvj77m.1.
